# Neurometabolites and sport-related concussion: From acute injury to one year after medical clearance

**DOI:** 10.1016/j.nicl.2020.102258

**Published:** 2020-04-22

**Authors:** Nathan W. Churchill, Michael G. Hutchison, Simon J. Graham, Tom A. Schweizer

**Affiliations:** a Keenan Research Centre of the Li Ka Shing Knowledge Institute at St. Michael's Hospital, Toronto, ON, Canada; b Neuroscience Research Program, St. Michael's Hospital, Toronto, ON, Canada; c Faculty of Kinesiology and Physical Education, University of Toronto, ON, Canada; d Department of Medical Biophysics, University of Toronto, Toronto, ON, Canada; e Physical Sciences Platform, Sunnybrook Research Institute, Sunnybrook Health Sciences Centre, Toronto, ON, Canada; f Faculty of Medicine (Neurosurgery) University of Toronto, Toronto, ON, Canada; g The Institute of Biomaterials & Biomedical Engineering (IBBME) at the University of Toronto, Toronto, ON, Canada

**Keywords:** Concussion, Brain injury, Neurometabolite, Svs, Dti, Bold fmri

## Abstract

Sport-related concussion is associated with acute disturbances in neurometabolic function, with effects that may last weeks to months after injury. However, is presently unknown whether these disturbances resolve at medical clearance to return to play (RTP) or continue to evolve over longer time intervals. Moreover, little is known about how these neurometabolic changes correlate with other measures of brain structure and function. In this study, these gaps were addressed by evaluating ninety-nine (99) university-level athletes, including 33 with sport-related concussion and 66 without recent injury, using multi-parameter magnetic resonance imaging (MRI), which included single-voxel spectroscopy (SVS), diffusion tensor imaging (DTI) and resting-state functional MRI (fMRI). The concussed athletes were scanned at the acute phase of injury (27/33 imaged), medical clearance to RTP (25/33 imaged), one month post-RTP (25/33 imaged) and one year post-RTP (13/33 imaged). We measured longitudinal changes in N-acetyl aspartate (NAA) and myo-inositol (Ins), over the course of concussion recovery. Concussed athletes showed no significant abnormalities or longitudinal change in NAA values, whereas Ins was significantly elevated at RTP and one month later. Interestingly, Ins response was attenuated by a prior history of concussion. Subsequent analyses identified significant associations between Ins values, DTI measures of white matter microstructure and fMRI measures of functional connectivity. These associations varied over the course of concussion recovery, suggesting that elevated Ins values at RTP and beyond reflect distinct changes in brain physiology, compared to acute injury. These findings provide novel information about neurometabolic recovery after a sport-related concussion, with disturbances that persist beyond medical clearance to RTP.

## Highlights

• Analyzed neurometabolite changes associated with sport-related concussion.
• Followed concussed athletes from acute injury to one year after return to play (RTP).
• Myo-inositol was elevated at RTP but had resolved at one year post-RTP.
• Neurometabolite response was greater in athletes without history of concussion.
• Myo-inositol levels were significantly correlated with DTI and fMRI parameters.

## 1 Introduction

Concussion involves biomechanical forces acting on the brain, leading to behavioural disturbances, typically in the absence of overt anatomical injury. The diagnosis and clinical management of sport-related concussion involves brief assessments encompassing mental status, co-ordination, balance and symptoms (McCrory, et al., 2017). The subsequent determination of safe return-to-play (RTP) is primarily based on symptom resolution and the completion of a graded exercise protocol. However, there remains insufficient understanding of the timeline of physiological brain recovery and its relationship with time of RTP.

Since the seminal animal work of Giza and Hovda (Giza and Hovda, 2001), it has been established that neurometabolic disturbances are a key component of concussion pathophysiology. In this domain, magnetic resonance single-voxel spectroscopy (SVS) has been a critical tool providing benchmarks of normal and disturbed neurometabolic function (Dimou and Lagopoulos, 2014). Studies have typically focused on metabolites that are both robustly detected and commonly associated with brain health. This most frequently includes N-acetyl aspartate (NAA), a marker of cell integrity and mitochondrial function; and myo-inositol (Ins), an osmolyte; along with creatine (Cr) and choline (Cho) which are typically used as reference standards (Blüml and Brooks, 2006; Lin, et al., 2012). Using SVS techniques, researchers have provided evidence that concussion creates a period of neurometabolic imbalance in the weeks following injury (Vagnozzi, et al., 2008; Vagnozzi, et al., 2007). During this time, abnormal levels of cerebral metabolites are associated with greater brain vulnerability.

Within the first week after a sport-related concussion, studies most commonly report NAA-related decreases relative to controls (Henry, et al., 2010; Henry, et al., 2011; Vagnozzi, et al., 2008). A few studies have reported resolution of neurometabolic disturbances within approximately one month post-injury (Vagnozzi, et al., 2010; Vagnozzi, et al., 2008); others have shown effects lasting weeks to months beyond this time, including persistent alterations in NAA – both increased and decreased relative to controls – along with the delayed emergence of elevated Ins values (Henry, et al., 2011; Vagnozzi, et al., 2013). There is also evidence that the trajectory of neurometabolic recovery may be significantly altered among athletes with a history of concussion (Johnson, et al., 2012a; Vagnozzi, et al., 2008). Overall, these studies are suggestive of neurometabolic disturbances that last beyond the initial symptomatic phase of injury, albeit with substantial variability in time to resolution. To date, no SVS studies have specifically examined changes relative to RTP. In addition, there has been limited research examining the relationship between SVS measures of neurometabolites and other MRI measures of brain structure and function post-injury, which may help to better understand the disturbances in brain physiology that accompany neurometabolic dysregulation (Chamard, et al., 2013).

In this study, we addressed this gap in knowledge by acquiring multi-parameter MRI data from concussed athletes, imaged longitudinally from the acute phase of injury (1 to 7 days post-injury) to one year post-RTP, along with a cohort of control athletes imaged prior to the start of their athletic season. This included SVS measures of neurometabolites within the primary motor cortex, as this area has previously been shown to be vulnerable to injury in sport-related concussion (Chamard, et al., 2013; Henry, et al., 2010; Henry, et al., 2011). In addition, we assessed white matter microstructure using diffusion tensor imaging (DTI) and resting-state brain function using blood-oxygenation-level dependent functional MRI (BOLD fMRI). The effects of concussion were evaluated longitudinally by modeling changes in neurometabolite ratios over time, relative to acute injury. The longitudinal models also accounted for the effects of history of concussion and time since injury, as these have been identified as important factors modifying neurometabolic response after a concussion. In an additional set of analyses, we evaluated correlations between neurometabolites values and other measures of brain physiology for both control and concussed groups, including DTI measures of white matter microstructure and BOLD fMRI measures of resting-state functional connectivity.

## 2 Methods

### 2.1 Study Participants

Ninety-nine (99) athletes participated in the study, which was carried out in accordance with the recommendations of the Canadian Tri-Council Policy Statement 2 (TCPS2) and with approval of the research ethics boards at the University of Toronto and St. Michael's Hospital. Thirty-three (33) concussed athletes were recruited consecutively from university-level sport teams at a single institution (including volleyball, hockey, soccer, football, rugby, basketball, lacrosse and water polo; see Appendix-A for athlete numbers by sport) through the academic sport medicine clinic, following concussion diagnosis. Diagnosis was determined by a staff physician following a sustained direct or indirect contact to the head with signs and/or symptoms as per the Concussion in Sport Group guidelines (McCrory, et al., 2017). Magnetic resonance imaging (MRI), including spectroscopy, was conducted at (1) the acute phase of injury (ACU; 1 to 7 days post-injury); (2) medical clearance to RTP (RTP); (3) one month post-RTP (1MO); and (4) one year post-RTP (1YR). Within the longitudinal study, some of the concussed athletes did not complete all imaging sessions. The number of participants retained at each time point was: ACU (27/33), RTP (25/33), 1MO (25/33) and 1YR (13/33). Attrition was not significantly related to demographic variables (age, sex, concussion history) or initial symptom severity, based on Spearman correlations with adjustment for multiple comparisons at a False Discovery Rate (FDR) of 0.05 across time points; however, longer time to RTP was related to greater attrition at RTP (ρ=0.498, *p* = 0.004) and at 1MO (ρ=0.439, *p* = 0.014).

As a control group, sixty-six (66) athletes without recent concussion were also consecutively recruited and imaged at the start of their competitive season. This included athletic controls with a prior history of concussion (HOC) as a comparison group, as a substantial proportion of concussed athletes also had HOC; all controls with HOC were imaged more than 9 months since last concussion. All athletes in the study completed baseline assessments with the Sport Concussion Assessment Tool (SCAT) (Echemendia, et al., 2017; Guskiewicz, et al., 2013) before the beginning of their athletic seasons. Athletes diagnosed with a concussion also completed SCAT assessments at acute injury and at time of RTP.

### 2.2 Magnetic Resonance Imaging

Athletes were imaged at St. Michael's Hospital using a research-dedicated MRI system operating at 3 Tesla (Magnetom Skyra, Siemens, Erlangen, Germany) with the standard multi-channel head receiver coil. Structural imaging included three-dimensional T1-weighted Magnetization Prepared Rapid Acquisition Gradient Echo imaging (MPRAGE: inversion time (TI)/echo time (TE)/repetition time (TR) = 1090/3.55/2300 ms, flip angle (FA) = 8°, 192 sagittal slices with field of view (FOV) = 240 × 240 mm, 256 × 256 pixel matrix, 0.9 mm slice thickness, 0.9 × 0.9 mm in-plane resolution, with bandwidth (BW) = 200 Hertz per pixel (Hz/px)), fluid attenuated inversion recovery imaging (FLAIR: TI/TE/TR = 1800/387/5000 ms, 160 sagittal slices with FOV=230 × 230 mm, 512 × 512 matrix, 0.9 mm slice thickness, 0.4 × 0.4 mm in-plane resolution, BW=751 Hz/px) and susceptibility-weighted imaging (SWI: TE/TR = 20/28 ms, FA = 15°, 112 axial slices with FOV=193 × 220 mm, 336 × 384 matrix, 1.2 mm slice thickness, 0.6 × 0.6 mm in-plane resolution, BW=120 Hz/px). To rule out potential structural abnormalities, structural scans were reviewed in a 2-step procedure, with initial inspection by a certified MRI technologist during the imaging session and later review by a neuroradiologist with clinical reporting if abnormalities were identified. No abnormalities (white matter hyper-intensities, contusions, micro-hemorrhage, or statistical outliers) were found among the concussed athletes and controls in this study.

Single voxel spectroscopy (SVS): data were acquired for two regions of interest, placed sequentially at the left and right primary motor cortex controlling the hands, using stimulated echo acquisition mode (STEAM) for 2 cm isotropic voxels (TM/TE/TR = 10/30/2000 ms; bandwidth = 1200 Hz; FA = 40°; 100 acquisitions; 1024 points). Regions were placed on an AC-PC-oriented axial slice corresponding to the region of interest first and confirmed using coronal and axial views to ensure adequate distance from ventricles, fatty tissue, and bone. Processing and analysis were conducted using the TARQUIN software package with default preprocessing parameter settings for STEAM, to obtain relative metabolite concentration values. The study focused on relative metabolite expression of NAA and Ins using Cr as a reference peak, by analyzing ratios NAA/Cr and Ins/Cr. To better approximate statistical normality, the ratios were transformed into log-ratios and values averaged across left- and right-side motor cortex. To control for heavy distribution tails in log-ratios of NAA/Cr (skewness: −0.69, kurtosis: 10.32) and Ins/Cr (skewness: −0.24, kurtosis: 4.06), the values were windsorized at the 95th percentile (2-tailed), bringing values to better approximate normality for both NAA/Cr (skewness: 0.28, kurtosis: 3.58) and Ins/Cr (skewness: −0.20, kurtosis: 2.53). Given evidence that Cr may change after TBI (Vagnozzi, et al., 2013), we also verified results using an alternate Cho reference, analyzing log- ratios NAA/Cho and Ins/Cho.

Diffusion tensor imaging (DTI): diffusion weighted imaging was performed (66 axial slices with FOV=240 × 240 mm, 120 × 120 matrix, 2.0 mm slice thickness, 2.0 × 2.0 in-plane resolution, BW=1736 Hz/Px), consisting of 30 diffusion-weighting directions (TE/TR = 83/7800 ms, *b* = 700 s/mm^2^, with 9 b0 scans). The DTI data were processed using utilities from the fMRIB Software Library (FSL; https://fsl.fmrib.ox.ac.uk) and customized algorithms developed in the laboratory. The FSL *eddy* protocol was used to perform simultaneous correction of eddy currents and rigid-body head motion, FSL *bet* was used to mask out non-brain voxels, and FSL *dtifit* was used to calculate voxel-wise measures of fractional anisotropy (FA) and mean diffusivity (MD).

Co-registration of DTI maps to a common template was obtained using DTI-TK software with default parameter settings (dti-tk.sourceforge.net). The IXI Aging DTI Template 3.0 was used as an initial reference, and a cohort-specific athlete template was generated using control data from (Churchill, et al., 2019). We obtained a sample of 44 athletes that were not part of the present study and were fully balanced on sex and HOC (mean age: 19.8 ± 1.6 yrs.; 11 male without HOC, 11 male with HOC, 11 female without HOC, 11 female with HOC), as an unbiased reference template. For this group, a bootstrapped template was obtained with *dti_template_bootstrap*, affine alignment and template updating was done using *dti_affine_population* (3 iterations), then diffeomorphic alignment and template updating was done with *dti_diffeomorphic_population* (6 iterations). The transform from athletic template to MNI space was afterwards obtained using the IIT Human Brain Atlas’ mean tensor template by sequentially applying rigid (*dti_rigid_reg*), affine (*dti_affine_reg*) and diffeomorphic (*dti_diffeomorphic_reg*) registration steps. For all athletes in this study, transforms to the athlete group template were then obtained by sequentially applying rigid (*dti_rigid_reg*), affine (*dti_affine_reg*) and diffeomorphic (*dti_diffeomorphic_reg*) steps. After, the net transforms into MNI space were computed using *dfRightComposeAffine* and were applied to DTI parameter maps via *deformationScalarVolume*. During registration, images were resampled to 3 × 3 × 3 mm resolution, and a 6 mm FWHM 3D Gaussian smoothing kernel applied to reduce spatial noise (e.g., due to scanner noise, head motion and minor alignment errors). Analysis was performed within a mask of white matter regions, i.e., where FA>0.30 in the group template, with manual segmentation and exclusion of brain stem areas with substantial field inhomogeneity.

Functional MRI (fMRI): data were acquired via multi-slice T2*-weighted echo planar imaging (EPI: TE/TR =30/2000 ms, FA = 70°, 32 oblique-axial slices with FOV=200 × 200 mm, 64 × 64 matrix, 4.0 mm slice thickness with 0.5 mm gap, 3.125 × 3.125 mm in-plane resolution, BW=2298 Hz/px), producing a time-series of 195 images at each slice location. During fMRI, athletes were instructed to lie still with their eyes closed and to not focus on anything in particular. Processing and analysis were performed using the Analysis of Functional Neuroimages (AFNI) package (afni.nimh.nih.gov), FSL and customized algorithms developed in the laboratory. After discarding the first 4 vol to allow the fMRI signal to reach equilibrium, the processing included rigid-body motion correction (AFNI *3dvolreg*), removal of outlier scan volumes using the SPIKECOR algorithm (nitrc.org/projects/spikecor), slice-timing correction (AFNI *3dTshift*), spatial smoothing with a 6 mm Full Width at Half Maximum (FWHM) isotropic 3D Gaussian kernel (AFNI *3dmerge*) and regression of motion parameters and linear-quadratic trends as nuisance covariates. For motion parameter regression, principal component analysis (PCA) was performed on the six rigid-body movement parameters (consistently accounting for >85% of variance), and the first two PCs were used as nuisance regressors. To control for physiological noise, the data-driven PHYCAA+ algorithm (nitrc.org/projects/phycaa_plus) was used to down-weight areas with non-neural signal, followed by regression of signal originating from white matter (WM) and cerebrospinal fluid (CSF). The WM and CSF regressions were performed after spatial normalization, described below.

The fMRI data were co-registered to a common anatomical template using the FMRIB Software Library (FSL) package (https://fsl.fmrib.ox.ac.uk). The FSL *flirt* algorithm was used to compute the rigid-body transform of the mean functional volume for each athlete to their T1-weighted anatomical image, along with the 12-parameter affine transformation of the T1 image for each athlete to the MNI152 template. The net transform was applied to the functional imaging data, which was resampled at 3 mm x 3 mm x 3 mm resolution. To remove WM and CSF signal, subject T1-weighted images were segmented and co-registered to the MNI152 template using the *fslvbm* protocol (fsl.fmrib.ox.ac.uk/fsl/fslwiki/FSLVBM), which used *fast* to obtain partial volume segmentation maps of gray matter (GM), WM and CSF, followed by iterative applications of affine registration algorithm *flirt* and nonlinear registration algorithm *fnirt*, to obtain a symmetric, study-specific mean GM tissue template. The spatial transforms were subsequently used to obtain mean WM and CSF tissue templates, resampled into 3 mm x 3 mm x 3 mm resolution and a 6 mm FWHM isotropic 3D Gaussian smoothing kernel was applied. For WM, the brain mask *P*(*WM*≥*P*_95%_(*WM*) was obtained (i.e., voxels within the distribution 95th percentile) and a single spatial erosion performed (3 × 3 kernel, in-plane). Two mean seed time series were obtained by separately averaging over cerebral white matter voxels and averaging over brainstem white matter (as their time courses were substantially different). For CSF, the brain mask *P*(*CSF*)≥*P*_95%_(*CSF*) was obtained and manually edited into two separate masks of the lateral ventricles. Two mean seed time series were obtained by separately averaging over these two ventricular regions. The four physiological time series were then regressed from each voxel, for all study participants. We then obtained voxel-wise maps of functional connectivity (Fconn), by calculating a mean seed time course within left and right hemisphere hand motor regions obtained using the Brainnetome Atlas (BNA) (Fan, et al., 2016), i.e., regions #57 and #58 (precentral gyrus, area 4, upper limb region), with center of mass MNI coordinates (−26, −25, 63) and (34, −19, 59). Connectivity analysis was done within a mask of GM regions segmented using T1 data and left and right hemisphere maps subsequently averaged for each subject.

### 2.3 Participant demographics

The demographics for control and concussed athlete groups were reported, along with pre-season baseline SCAT symptoms (severity and total number of symptoms) for both groups. For concussed athletes, we also reported SCAT symptoms at acute injury and RTP. Furthermore, symptoms at acute injury and RTP were compared to concussed athletes’ pre-season baseline values using paired-measures Wilcoxon tests. Significant tests were reported after adjusting for multiple comparisons at an FDR of 0.05. Unless otherwise noted, group statistics were summarized by the median with upper and lower quartiles.

### 2.4 Longitudinal effects of concussion

To model longitudinal changes in neurometabolites in the presence of missing data (see **Section 2.1**), the effect of imaging session was estimated for each metabolite log-ratio in a linear mixed effects model (LMM), with fixed effects of imaging session (RTP, 1MO, 1YR) measured relative to ACU and with subject-specific random-effects intercepts. Given the literature evidence for effects of HOC and time post-injury on neurometabolite response, we also examined how of these variables influenced neurometabolite recovery by including them as interaction terms at each time point post-injury (i.e., measuring the simple effects on concussed athletes). We modeled HOC as a binary variable; for the acute time point, we modeled days post-injury (dACU), centered relative to the mean (5 days); for post-acute time points, we modeled days to RTP (dRTP), where to control for heavy distribution tails (skewness: 1.79, kurtosis: 5.78) we applied an inverse empirical distribution transform before mean-centering. The models were fitted using the Matlab R2017b *fitlme* package (The MathWorks, Natick MA) with full covariance estimation using Cholesky parameterization. Analysis was done in a bootstrap resampling framework, where resampling units consisted of all spectroscopy data for a given participant (1000 iterations). We subsequently reported fixed-effect coefficients *b* with 95% confidence intervals (95%CIs), standardized effect sizes based on the bootstrap ratio (BSRs; mean / standard error) and empirical p-values, with significant tests identified at an FDR of 0.05. We also plotted the mean neurometabolite response at each time point, with bootstrapped 95%CIs of the mean, along with the mean and 95%CI of control data for comparison. In addition, we conducted cross-sectional analyses at each imaging session (ACU, RTP, 1MO, 1YR), comparing concussed athletes to athletic controls. Given the anticipated effects of HOC on neurometabolite response, we separately compared concussed athletes without HOC to controls without HOC and concussed athletes with HOC to controls with HOC, using 2-sample bootstrap analyses. For cross-sectional analyses, mean effects and 95% CIs were reported, along with BSRs and p-values, and significant tests were identified at an FDR of 0.05.

To mitigate bias and loss of efficiency due to missing data, bootstrap distributions on both the mean neurometabolite response and cross-sectional comparisons of concussed and control groups were combined with multiple imputation using the “Boot MI” approach of (Schomaker and Heumann, 2018): during resampling, bootstrap samples were drawn from the full dataset (including missing data) and for each sample, imputation was done *M* = 10 times to generate 10 coefficient estimates, which were averaged to obtain a point estimate. The set of coefficient point estimates were treated as a conventional bootstrap empirical distribution, from which summary statistics were calculated. Imputation was done using the fitted LMM to generate simulated metabolite log-ratio values.

### 2.5 Associations with other MRI parameters

For neurometabolites showing significant longitudinal effects, we examined correlations with other MRI parameters including FA, MD and Fconn. This was characterized separately for controls and for concussed athletes at each time point post-injury, to determine whether concussed athletes show distinct associations between neurometabolites and other MRI measures of brain physiology, compared to controls, and whether these relationships change over the course of concussion recovery. Analyses were performed on whole-brain maps to examine whether SVS measurements within the motor cortex were sensitive to spatially distributed changes in brain physiology. Voxel-wise non-parametric Spearman correlations were calculated between SVS measures and other MRI parameters, bootstrapped empirical distributions were obtained on the correlation coefficient values (1000 iterations) and significant brain regions were identified by applying a voxelwise threshold at *p* = 0.005, followed by cluster-size thresholding at an adjusted *p* = 0.05, using AFNI *3dFWHMx* to estimate the spatial smoothness of maps, followed by AFNI *3dClustSim* to obtain the minimum cluster size threshold. To mitigate bias and efficiency loss due to missing data, imputation was performed on imaging data prior to correlation analyses using the non-parametric SOFT-IMPUTE approach (Mazumder, et al., 2010), with model fitting details provided in Appendix-C.

For Fconn, we also evaluated whether there was an association between regional connectivity strength (i.e., to the motor cortex seed) and strength of neurometabolite effects among concussed athletes. This was determined by obtaining at each voxel the mean connectivity value of athletic controls *ρ_ctl_* and the Spearman correlation between neurometabolite and Fconn values for concussed athletes *ρ_SVS_,F_conn_*. We then calculated the Spearman correlation between paired voxel values *ρ_ctl_* and *ρ_SVS_,F_conn_*, with bootstrap resampling performed to obtain 95% CIs and p-values.

## 3 Results

### 3.1 Participant demographics

Table 1 summarizes demographic and clinical data for the control and concussed groups (see Table S1 of Appendix-A for sport numbers). For controls, athletes with HOC did not have significantly elevated symptoms compared to those without HOC (*z* = 0.16 and *p* = 0.875). For the concussed athletes, symptoms at acute injury were significantly elevated relative to the controls and their own baseline, for total symptoms and symptom severity (*z* ≥ 3.35 and *p* ≤ 0.001, all tests). In contrast at RTP, scores for concussed athletes had recovered and become slightly lower compared to both baseline and athletic controls (*z*≤−2.63 and *p* ≤ 0.008, all tests). For concussed athletes with HOC, symptoms were not significantly higher compared to those without prior HOC at any of the assessment time points (*z* ≤ 1.27 and *p* ≥ 0.205, all tests), nor was time to RTP significantly prolonged in this group (*z* = 0.26, *p* = 0.794). Control athletes with prior HOC reported a median of 2 prior concussions with IQR [1, 2], that occurred a median of 31 months [12, 56] prior to imaging (27/31 occurring >1 year prior to imaging). Concussed athletes with prior HOC reported a median of 2 prior concussions [1, 2] occurring a median of 24 months [10, 69] before their most recent injury, indicating comparable HOC demographics for the concussed and control cohorts.

**Table 1:** Demographic data for athletes with concussion and control athletes, along with symptom and cognitive scores, based on the sport concussion assessment tool (SCAT3). The SCAT3 scores are represented as the median [Q1, Q3]. ‘*’ indicates a significant difference in scores for the symptomatic time-point (SYM), relative to all other groups.

	Control	Concussion		
**Age (mean ± SD)**	20.5 ± 1.7		20.3 ± 2.0	
**Female**	36/66 (55%)		17/33 (52%)	
**Previous concussion**	31/66 (47%)		19/33 (58%)	
**Collision sports**	20/66 (30%)		22/33 (67%)	
**Days to RTP**	–		22 [14, 95]	
		**Baseline**	**Acute**	**RTP**
**Total Symptoms**	2 [0, 5]	2 [1, 5]	8 [4, 15]*	0 [0, 2]
**Symptom Severity**	3 [0, 8]	3 [1, 8]	9 [4, 31]*	0 [0, 2]

### 3.2 Control neurometabolic data

Within the control group, there was no significant effect of history of concussion on metabolite log-ratios, both for NAA/Cr (*b* = 0.046, 95%CI: −0.056, 0.149; BSR=0.90; *p* = 0.370) and for Ins/Cr (*b* = 0.086, 95%CI: −0.0277, 0.200; BSR=1.51; *p* = 0.135). Supplemental testing of demographic and clinical factors (age, sex, collision sport, symptoms) did not find any significant effects (|BSR|≤1.59, *p* ≥ 0.117 for all tests). Moreover, among the control athletes with history of concussion, there was no significant effect of total number of concussions or time since their last injury (|BSR|≤1.93, *p* ≥ 0.065 for all tests).

### 3.3 Longitudinal effects of concussion

**Fig. 1:**
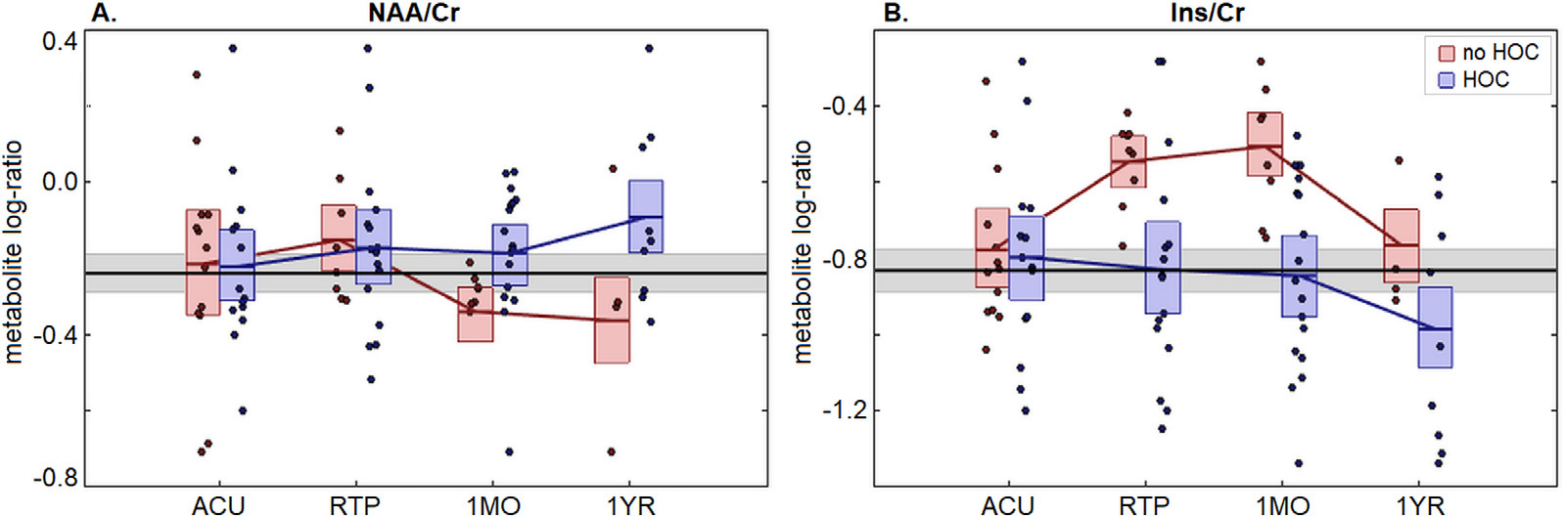
**plot depicting longitudinal changes in log-ratios of (A) NAA/Cr and (B) Ins/Cr for concussed athletes**. Log-ratio values of individual athletes are plotted for each imaging session (ACU, RTP, 1MO, 1YR), with distributions plotted separately for groups with and without history of concussion (HOC). For the distribution plots, horizontal red/blue lines denote group means and boxes indicate bootstrapped 95% confidence intervals for the means; distribution means are connected between sessions by solid red/blue lines. The mean log-ratio value for controls is plotted as the thick horizontal black line, with corresponding 95% confidence interval shaded in gray.

For concussed athletes, the longitudinal changes in NAA/Cr and Ins/Cr log-ratios are summarized in Table 2, with differences relative to athletic controls reported in Table 3 and log-ratio values plotted in Fig. 1 (very similar results were found for NAA/Cho and Ins/Cho, reported Appendix-B). Examining the longitudinal changes in NAA/Cr values, relatively wide confidence bounds were observed, with effects that did not attain statistical significance for any of the post-injury time points. For HOC, we identified increased NAA/Cr values at 1MO and effects of time post-injury at ACU at a nominal *p*<0.05 threshold, but effects were non-significant at an adjusted FDR of 0.05. No significant differences were found when concussed athletes were compared to controls.

**Table 2:** Longitudinal effects of concussion on NAA/Cr and Ins/Cr. The table reports coefficients of fixed-effect *b*, 95% confidence intervals (95% CIs), bootstrap ratios (BSRs) and p-values. Longitudinal tests compare imaging sessions (RTP, 1MO, 1YR) to ACU and interactions with history of concussion (HOC), days post injury (dACU) and days to RTP (dRTP). Significant tests, after adjusting for multiple comparisons at FDR of 0.05 are shown in bold.

	NAA/Cr				Ins/Cr			
	*b*	95%CI	BSR	*p*	*b*	95%CI	BSR	*p*
ACU	–	–	–	–	–	–	–	–
RTP	0.095	[−0.039, 0.276]	1.43	0.146	0.230	[0.073, 0.37]	3.08	**0.009**
1MO	−0.101	[−0.235, 0.058]	−1.23	0.220	0.276	[0.108, 0.437]	3.44	**0.004**
1YR	−0.121	[−0.402, 0.166]	−0.91	0.332	0.021	[−0.234, 0.324]	0.22	0.740
ACU:HOC	0.034	[−0.111, 0.195]	0.51	0.606	−0.031	[−0.222, 0.149]	−0.40	0.684
RTP:HOC	−0.034	[−0.214, 0.097]	−0.70	0.490	−0.278	[−0.43, −0.126]	−3.58	**<0.001**
1MO:HOC	0.151	[0.026, 0.272]	2.39	0.024	−0.347	[−0.528, −0.194]	−4.28	**<0.001**
1YR:HOC	0.265	[−0.074, 0.596]	1.51	0.114	−0.225	[−0.584, 0.078]	−1.39	0.130
ACU:dACU	0.061	[0.003, 0.117]	2.12	0.042	−0.034	[−0.118, 0.026]	−1.38	0.166
RTP:dRTP	−0.001	[−0.084, 0.088]	0.03	0.986	−0.004	[−0.1, 0.113]	−0.08	0.902
1MO:dRTP	−0.082	[−0.163, 0.014]	−1.69	0.094	0.005	[−0.089, 0.104]	−0.01	0.920
1YR:dRTP	−0.121	[−0.267, 0.098]	−1.15	0.210	−0.032	[−0.332, 0.217]	−0.33	0.646

**Table 3:** Cross-sectional comparison of concussed NAA/Cr and Ins/Cr to athletic controls. The table reports mean effects, 95% confidence intervals (95% CIs), bootstrap ratios (BSRs) and p-values. Tests compare imaging sessions (ACU, RTP, 1MO, 1YR), for athletes with and without history of concussion (HOC).

		NAA/Cr				Ins/Cr			
		*mean*	95%CI	BSR	*p*	*mean*	95%CI	BSR	*p*
ACU	(no HOC)	0.021	[−0.156, 0.200]	0.23	0.806	0.052	[−0.098, 0.219]	0.63	0.529
	(HOC)	0.018	[−0.116, 0.154]	0.26	0.800	0.031	[−0.117, 0.183]	0.40	0.699
RTP	(no HOC)	0.087	[−0.061, 0.230]	1.19	0.228	0.286	[0.156, 0.425]	4.05	**<0.001**
	(HOC)	0.068	[−0.068, 0.206]	0.97	0.348	−0.001	[−0.159, 0.170]	−0.01	0.931
1MO	(no HOC)	−0.099	[−0.236, 0.038]	−1.43	0.158	0.326	[0.187, 0.471]	4.24	**<0.001**
	(HOC)	0.054	[−0.076, 0.181]	0.83	0.390	−0.014	[−0.161, 0.153]	−0.18	0.828
1YR	(no HOC)	−0.122	[−0.285, 0.038]	−1.46	0.134	0.066	[−0.078, 0.234]	0.83	0.415
	(HOC)	0.145	[0.009, 0.274]	2.09	0.036	−0.150	[−0.299, 0.005]	−1.95	0.055

Examining the longitudinal change in Ins/Cr values relative to ACU, increases were observed at RTP and 1MO that were significant at an FDR of 0.05, followed by a return to non-significance at 1YR (Table 2). However, we also observed a significant, opposite effect of HOC on Ins/Cr values at both RTP and 1MO. As shown in Fig. 1, although concussion was associated with transient increases in Ins/Cr, the effect was largely absent in athletes who had previous concussions. In general, the effects of time post-injury were non-significant. The observed elevations in Ins/Cr values are supported by comparisons to athletic controls, as concussed athletes without HOC were significantly different from controls at RTP and 1MO at an FDR of 0.05, whereas those with HOC were not significantly different.

### 3.4 Associations with other MRI parameters

**Fig. 2:**
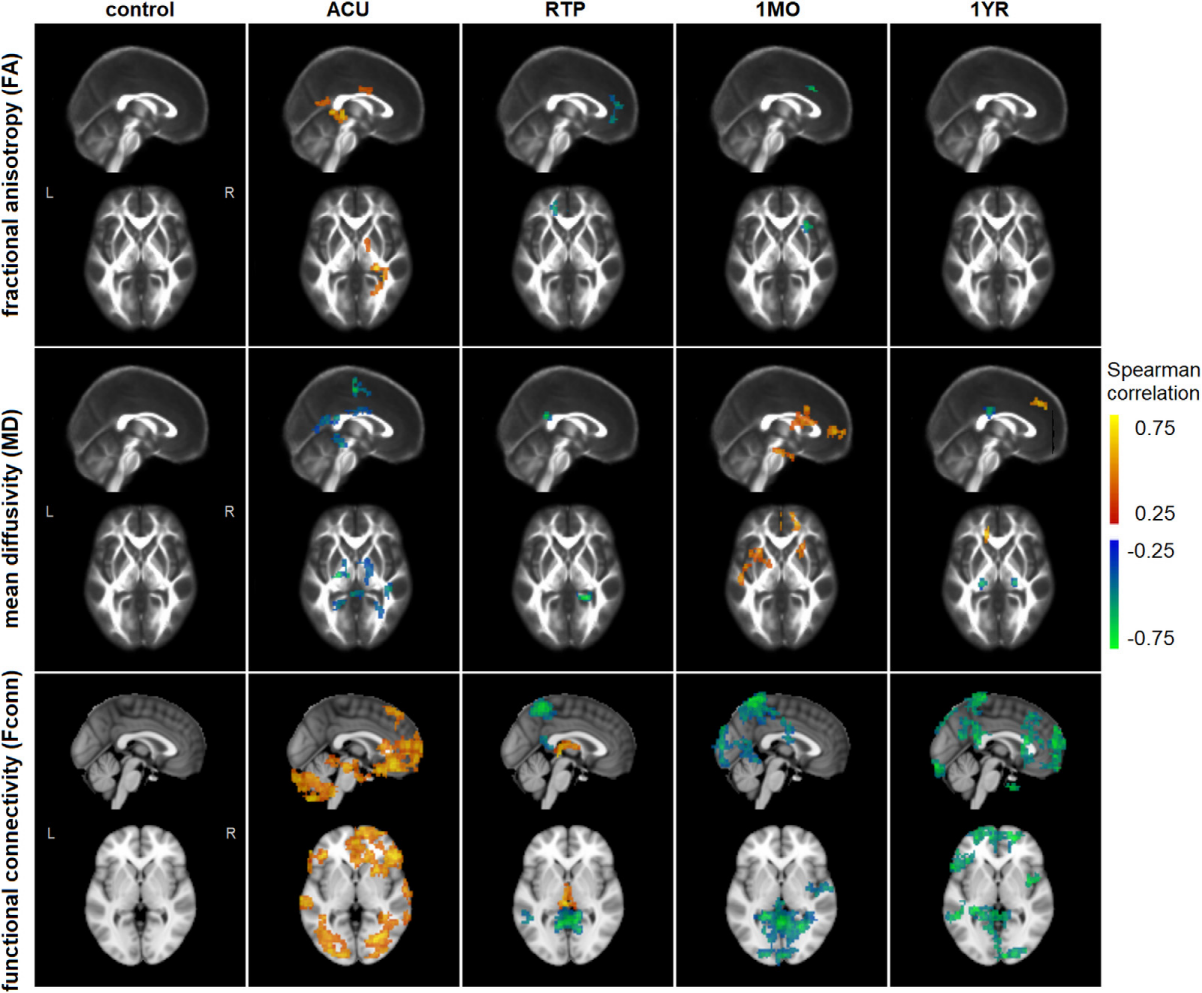
**associations between Ins/Cr log-ratios and other MRI parameters, including white matter fractional anisotropy (FA) and mean diffusivity (MD), along with functional connectivity to the primary motor cortex (Fconn)**. Brain maps depict areas of significant Spearman correlation, adjusted for multiple comparisons. Results are shown for athletic controls and concussed athletes at each imaging session (ACU, RTP, 1MO, 1YR). Slices are shown as maximum intensity projections (MIPs) in the axial and sagittal planes (MNI coordinates *x* = 0, *z* = 0).

Fig. 2 plots the results of voxelwise analyses correlating Ins/Cr values with white matter FA and MD, along with gray matter functional connectivity Fconn. For FA, no significant associations were observed with Ins/Cr values among athletic controls, whereas significant associations were seen for concussed athletes (clusters summarized in Table 4). For concussed athletes, the most spatially extensive associations were at ACU, with positive correlations in the internal capsule, posterior thalamic radiation and corpus callosum. An opposite relationship was observed at later time points, with negative correlations at RTP within the anterior corona radiata and at 1MO within the superior corona radiata, but effects were non-significant at 1YR.

**Table 4:** Cluster report for associations between Ins/Cr log-ratios and regional fractional anisotropy (FA) values, displayed in Fig. 2. Centers of mass are in MNI coordinates and brain region based on nearest labelled gray matter region in the Johns Hopkins University (JHU) atlas.

	Cluster	Center of mass			Brain region	Cluster size (mm^3^)	Peak value (correlation)
**ACU**	1	30	−36	0	Internal capsule (retrolent. part) R	1728	0.67
	2	30	−57	15	Posterior thalamic radiation R	702	0.52
	3	15	−3	30	Body of corpus callosum R	648	0.49
**RTP**	1	−18	45	9	Anterior corona radiata L	918	−0.61
**1MO**	1	33	21	33	Superior corona radiata R	648	−0.70

For MD, we also observed non-significant correlations with Ins/Cr among athletic controls, whereas significant associations were seen for concussed athletes (clusters summarized in Table 5). For concussed athletes, the most spatially extensive associations were again at ACU, with negative correlations in the corona radiata, corpus callosum, sagittal stratum and posterior thalamic radiation. Negative correlations were sparser at RTP and limited to the corpus callosum, with positive correlations appearing at 1MO in the corona radiata and sagittal stratum. A mixture of effects was later seen at 1YR, with mainly negative correlations in posterior and superior corona radiata but also positive correlations in anterior corona radiata.

**Table 5:** Cluster report for associations between Ins/Cr log-ratios and regional mean diffusivity (MD) values, displayed in Fig. 2. Centers of mass are in MNI coordinates and brain region based on nearest labelled gray matter region in the Johns Hopkins University (JHU) atlas.

	Cluster	Center of mass			Brain region	Cluster size (mm^3^)	Peak value (correlation)
**ACU**	1	−18	−15	57	Superior corona radiata L	1593	−0.69
	2	15	−9	30	Body of corpus callosum R	1458	−0.49
	3	42	−36	−9	Sagittal stratum R	1215	−0.58
	4	−21	−51	18	Splenium of corpus callosum L	999	−0.55
	5	3	−39	18	Splenium of corpus callosum R	918	−0.57
	6	33	−60	12	Posterior thalamic radiation R	783	−0.49
**RTP**	1	18	−45	24	Splenium of corpus callosum R	1161	−0.74
**1MO**	1	−27	9	21	Superior corona radiata L	2241	0.59
	2	18	45	3	Anterior corona radiata R	1215	0.65
	3	24	15	15	Anterior corona radiata R	918	0.56
	4	−42	−15	−18	Sagittal stratum L	864	0.60
**1YR**	1	−24	−27	30	Posterior corona radiata L	864	−0.64
	2	24	−24	30	Superior corona radiata R	702	−0.63
	3	−15	36	39	Anterior corona radiata L	594	0.69

For Fconn, the athletic controls showed no significant correlations with Ins/Cr, whereas significant associations are seen for concussed athletes (clusters summarized in Table 6). Conversely, concussed athletes had uniformly positive significant correlations at ACU, localized primarily within frontal and cerebellar regions. These effects had largely dissipated at later imaging sessions, with negative correlations appearing in parietal regions at RTP and becoming more spatially extensive at 1MO. At 1YR, negative correlations were still present, but now localized more in frontal regions. The correlations between Fconn and Ins at ACU tended to be greater in areas with lower connectivity to the motor cortex, with a Spearman correlation of ρ=−0.563 (95%CI: −0.674, −0.064; *p* = 0.028), whereas this relationship was weaker at later imaging time points, with |ρ|≤0.319, *p* ≥ 0.051.

**Table 6:** Cluster report for associations between Ins/Cr log-ratios and regional functional connectivity(Fconn) values, displayed in Fig. 2. Centers of mass are in MNI coordinates and brain region based on nearest labelled gray matter region in the automated anatomical labeling (AAL) atlas.

	Cluster	Center of mass			Brain region	Cluster size (mm^3^)	Peak value (correlation)
**ACU**	1	9	57	3	Superior frontal (medial) R	13,041	0.66
	2	33	−69	−45	Cerebellum crus2 R	9747	0.71
	3	42	30	−15	Inferior orbitofrontal R	9612	0.71
	4	−33	−75	−36	Cerebellum crus2 L	9396	0.66
	5	6	0	51	Supplementary motor area R	2970	0.63
	6	−63	−24	−18	Inferior temporal L	2079	0.68
	7	−45	36	15	Inferior orbitofrontal L	1809	0.69
	8	66	−12	−24	Middle temporal R	1728	0.59
	9	−57	24	9	Inferior frontal (triang. part) L	1242	0.63
	10	66	−45	−9	Inferior temporal R	972	0.56
**RTP**	1	3	−51	57	Precuneus R	11,313	−0.76
	2	−3	−21	9	Thalamus L	2349	0.71
	3	−51	−45	15	Middle temporal L	1026	−0.56
**1MO**	1	6	−51	60	Precuneus R	19,629	−0.76
	2	3	−93	6	Calcarine R	3537	−0.65
	3	−9	−63	3	Lingual L	3186	−0.58
	4	51	−9	27	Postcentral R	2781	−0.60
	5	−21	−90	30	Superior occipital L	1431	0.64
	6	18	−66	12	Calcarine R	1188	−0.53
**1YR**	1	−15	60	0	Superior frontal (medial) L	6858	−0.73
	2	−48	24	21	Inferior frontal (triang. part) L	5427	−0.74
	3	−9	−42	69	Precuneus L	5400	−0.80
	4	−54	−42	24	Superior temporal L	3132	−0.72
	5	21	−84	−21	Cerebellum crus1 R	3051	−0.79
	6	21	57	21	Superior frontal R	2376	−0.76
	7	−9	−60	30	Precuneus L	1755	−0.64
	8	45	3	−42	Inferior temporal R	1080	−0.81
	9	−3	−81	45	Precuneus L	1053	−0.63

Table 7 summarizes the correlations between Ins/Cr values and other MRI parameters (FA, MD, Fconn), averaged over significant voxels. This includes Spearman correlations between MRI parameters and Spearman partial correlations after controlling for HOC, showing to what extent the relationships between MRI parameters are driven by HOC. Both correlations and partial correlations have moderate-to-high values for all analyses, with bootstrapped 95%CIs that do not enclose zero. For all MRI parameters, as expected, controlling for HOC had the greatest effect on correlations at RTP followed by 1MO, the time points when Ins/Cr response is most affected by HOC. The FA response at RTP was most affected, with an average 32.7% reduction in correlation strength, while MD showed a 19.6% reduction and Fconn had the weakest effect with a 15.9% reduction. Only FA shows a reduction at a nominal *p*<0.05 threshold, although effects were non-significant at an FDR of 0.05.

**Table 7:** Correlation statistics for the relationship between Ins/Cr log-ratios and other MRI parameters, including white matter fractional anisotropy (FA) and mean diffusivity (MD), along with functional connectivity to the primary motor cortex (Fconn), averaging over significant brain regions as reported in Table 4, Table 5, Table 6. Values include Spearman correlations (*corr*) and partial correlations (*pcorr*) after adjusting for history of concussion (HOC), along with the change in absolute correlation strength |*pcorr*|-|*corr*|. Statistics include bootstrapped 95% confidence intervals (95%CIs) and empirical p-values on the change values.

		*corr*	95%CI	*pcorr*	95%CI	|*pcorr*|-|*corr*|	95%CI	*p*
FA	ACU	0.693	[0.475, 0.839]	0.693	[0.482, 0.848]	0.001	[−0.083, 0.074]	0.481
	RTP	−0.648	[−0.832, −0.372]	−0.435	[−0.738, −0.053]	−0.212	[−0.462, −0.033]	0.005
	1MO	−0.718	[−0.892, −0.472]	−0.705	[−0.865, −0.478]	−0.014	[−0.123, 0.105]	0.374
	1YR	–	–	–	–	–	–	–
MD	ACU	−0.680	[−0.828, −0.453]	−0.696	[−0.842, −0.479]	0.016	[−0.057, 0.103]	0.297
	RTP	−0.647	[−0.831, −0.393]	−0.517	[−0.780, −0.193]	−0.127	[−0.325, 0.037]	0.071
	1MO	0.645	[0.370, 0.829]	0.540	[0.246, 0.766]	−0.104	[−0.280, 0.045]	0.075
	1YR	−0.611	[−0.835, −0.327]	−0.579	[−0.823, −0.247]	−0.032	[−0.199, 0.077]	0.339
Fconn	ACU	0.765	[0.536, 0.913]	0.767	[0.508, 0.923]	0.002	[−0.070, 0.061]	0.419
	RTP	−0.618	[−0.819, −0.306]	−0.520	[−0.792, −0.186]	−0.098	[−0.283, 0.055]	0.123
	1MO	−0.752	[−0.892, −0.552]	−0.681	[−0.848, −0.390]	−0.072	[−0.266, 0.051]	0.153
	1YR	−0.760	[−0.907, −0.530]	−0.728	[−0.894, −0.498]	−0.032	[−0.159, 0.055]	0.278

## 4 Discussion

Neurometabolic disturbances are a common sequela of concussion and previous studies have identified disturbances in the first days after injury, with effects that may last weeks to months afterwards. This SVS study expands on prior literature by focusing on changes within the hand region of the primary motor cortex from acute injury to RTP, with follow-up at one month and one year post-RTP. Our key finding was that Ins shows ongoing alterations at the time of medical clearance to RTP, with dissipation occurring between one month and one year afterwards. The Ins response to concussion was also substantially attenuated for athletes with HOC, whereas time post-injury did not have a significant effect. Subsequent analyses identified correlations between Ins values and other MRI parameters among concussed athletes, including FA and MD of white matter, along with Fconn of the motor cortex. These findings suggest that differing Ins levels among concussed athletes are associated with alterations in tissue microstructure and brain function during the course of concussion recovery.

Our analyses showed an absence of longitudinal changes in NAA during recovery, or of significant disturbances relative to athletic controls. Prior longitudinal studies have reported variable effects: one study found no significant effects in the corpus callosum over the span of 3 days to 2 months (Chamard, et al., 2012); others reported reduced NAA in frontal white matter at 3 days, with recovery complete by 30 days (Vagnozzi, et al., 2010; Vagnozzi, et al., 2008); and another found persistent NAA elevations within motor and prefrontal gray matter at 6 days and 6 months post-injury (Henry, et al., 2011). NAA is one of the most abundant metabolites in the brain, and is linked to energy metabolism and mitochondrial function (Moffett, et al., 2013). The absence of significant findings in this study may be due to primary neurometabolic disturbances showing rapid resolution (Giza and Hovda, 2014), as we identified a nominally significant effect of days post-injury within the acute phase of injury. Alternatively, it may be due to the selected region(s) of interest. In SVS studies examining multiple brain regions, there is evidence of substantial spatial variability in direction and magnitude of neurometabolic disturbances (Henry, et al., 2010; Henry, et al., 2011; Johnson, et al., 2012a), which is potentially due to spatial variations in injury-related biomechanical forces (Tarlochan, 2013; Viano, et al., 2005).

We observed longitudinal changes in Ins over the course of clinical recovery, with significant elevations at RTP and one month afterwards. Although few concussion studies have examined Ins, one study found no significant Ins effects in the corpus callosum over the span of 3 days to 2 months (Chamard, et al., 2012), whereas another reported effects similar to those of the present work, with normal Ins values in the motor cortex at 6 days post-injury and elevated values at 6 months post-injury (Henry, et al., 2011). Ins is highly abundant in the brain, particularly within glial cells, and is thought to function primarily as an osmolyte, although it is also involved in synthesis of cell membranes and myelin (Haris, et al., 2011). As previously suggested by (Henry, et al., 2011), an absence of acute concussion effects on Ins may be due to the brain accumulating Ins to offset ionic imbalances at early injury (Lien, et al., 1990). However, the presence of a delayed increase in Ins suggests a pathophysiologic process that is distinct from the early neurometabolic cascade, which is thought to have largely resolved within one week post-injury (Giza and Hovda, 2014). This may be due to continuing accumulation of Ins, e.g., to offset persistent alterations in cell tonicity. Alternatively, as Ins is considered a marker of glial cell proliferation (Brand, et al., 1993), this may be due to delayed gliosis (Mannix, et al., 2014; Ojo, et al., 2016). Our results show that these effects are present at medical clearance to RTP and one month afterwards, although resolution occurs by one year post-RTP.

We also identified a significant effect of prior concussion history on Ins response, as athletes with HOC had a diminished Ins response at RTP and one month afterwards. These effects were seen despite non-significant differences in SCAT symptom scores, hence neurometabolic alterations due to HOC may be detected in the absence of measurable differences on standard clinical assessments. The effects of HOC on post-concussion response has been previously reported for NAA, although the specific effects have been variable. One study reported diminished effects of concussion on NAA for athletes with prior HOC (Johnson, et al., 2012a), while another reported prolonged concussion-related decreases in NAA (Vagnozzi, et al., 2008). The present findings are the first to show a moderating effect of HOC for Ins, as further evidence that concussions have a cumulative effect on neurometabolism. It is presently unclear whether this attenuation of Ins response among athletes with HOC represents impaired recovery after concussion, or an adaptive response to mitigate the neurodegenerative consequences of persistent glial activation (Witcher and Godbout, 2017). Future research will be critical to elucidate these details. Interestingly, despite the substantial variability in time to RTP, there was no significant effect of time post-injury on neurometabolite response. This is also consistent with prior literature (Johnson, et al., 2012a) and suggests that the concussion-related changes in Ins evolve over relatively long time intervals.

In the present study, we did not have longitudinal control data to assess normal intra-subject longitudinal variability, making it unclear whether concussion-related changes in neurometabolite levels exceed normal brain variability. However, based on prior publications (Bartha, et al., 2000; Brooks, et al., 1999; Geurts, et al., 2004) and standard formulae for propagation of error (Appendix-D), the estimated normal 95%CIs of variability for log-ratios are ± 0.145 for NAA/Cr and ± 0.196 for Ins/Cr. Hence, the elevations in Ins/Cr values at RTP and 1MO significantly exceed normal variability. However, these studies may under-estimate longitudinal variability in uninjured varsity athletes, as the demands of sport and academics may be associated with substantial physiological and psychological stressors, both of which can affect longitudinal changes in neurometabolites (Jung, et al., 2019; Karl and Werner, 2010; Moriguchi, et al., 2019). Hence there is a need for normative longitudinal athlete data, to more accurately determine whether the observed concussion-related neurometabolites changes exceed normal variations in brain physiology for this cohort.

To date, there has been limited research examining associations between SVS neurometabolites and other MRI measures of brain physiology in sport-related concussion. One study reported that Ins was negatively correlated with MD at 7 months post-injury, but the relationship with FA was non-significant (Chamard, et al., 2013). At present, it is unclear how these results relate to the present findings, as 7 months post-injury is (on average) midway between our imaging sessions at one month and one year post-RTP. At acute injury, higher Ins was correlated with higher FA and lower MD; this is consistent with the interpretation that elevated Ins in the brain offsets osmotic imbalance, as cellular edema is typically associated with lower FA and higher MD values (Assaf and Pasternak, 2008). Interestingly, we observe changes in the direction of correlation at RTP and afterwards, suggesting the delayed emergence of pathophysiological processes that are distinct from acute injury. In particular, elevated Ins was correlated with lower FA at RTP, and with both lower FA and higher MD at one month post-RTP. This is consistent with a hypothesis of glial proliferation, which would lead to both increased Ins (Brand, et al., 1993) and reduced FA / increased MD within white matter tracts (Budde, et al., 2011). However, we also note that the clusters showing significant FA and MD effects were spatially limited and showed substantial spatial variability across imaging time points. Additional research is therefore required to further evaluate these hypotheses about the cellular mechanisms that underlie long-term neurometabolic recovery.

To our knowledge, this is the first study linking SVS neurometabolites to fMRI functional connectivity, showing a significant relationship with brain function throughout the course of recovery. The spatially distributed patterns of significant correlations indicate that neurometabolic variations within the motor cortex are correlated with alterations in functional integration throughout the brain. Higher Ins was correlated with elevated Fconn at acute injury, with the greatest effects in regions of low intrinsic connectivity to the motor cortex. This may be interpreted as higher acute Ins levels reflecting adaptive or compensatory response at early injury; there is evidence that hyper-connectivity, particularly between functionally distinct modules, helps to sustain function after brain injury, albeit at the cost of reduced efficiency (Hillary, et al., 2015; Hillary and Grafman, 2017). Conversely, higher Ins was correlated with reduced Fconn at RTP and later imaging time points. This suggests that, beyond the acute window of injury, Ins is an indicator of disrupted long-range functional connectivity. Post-acute declines in functional connectivity have been previously reported (Johnson, et al., 2012b) and are potentially related to glial activation as previously discussed for the DTI results.

The Ins-related disturbances in functional connectivity of the motor cortex are highly relevant to the varsity athlete population, as effective motor control is needed to perform at the high levels required of both training and competition, and to avoid further concussive or subconcussive impacts. The alterations in connectivity to mainly frontal and cerebellar regions at acute injury and at one year post-RTP are consistent with changes in motor networks associated with adaptation and/or compensation in other neurological conditions, including TBI (Kasahara, et al., 2010), stroke (Park, et al., 2011; Wadden, et al., 2015) and multiple sclerosis (Pantano, et al., 2002; Saini, et al., 2004). Whereas at RTP and one month post-RTP, the precuneus most consistently shows altered connectivity. This is a densely-connected region that is involved in visuo-spatial processing during guidance of motor movements (Wenderoth, et al., 2005) but also in episodic memory (Dörfel, et al., 2009) and higher-level representations of self (Lou, et al., 2004). Hence, impaired connectivity of the motor cortex with this region may lead to subtle impairments in integrating multiple different information streams when guiding motor response. While the athletes in this study showed no deficits at RTP based on standard clinical evaluation, these findings point towards a potential vulnerability which should be assessed in future studies and suggest that Ins may be a useful correlate of functional brain changes.

The correlation analyses of Ins and other MRI parameters provide novel information about the physiological processes that underlie inter-individual variations in neurometabolite levels. Initial voxelwise analyses focused on group-level correlations among concussed athletes, without accounting for whether these effects are driven by specific demographic and clinical factors. Post-hoc analyses then examined the influence of HOC via partial correlations, given its impact on longitudinal Ins response during concussion recovery. Our findings show that HOC had the greatest effect at RTP but accounted for a relatively small amount of correlation between these parameters at this time. Therefore, it appears unlikely that the observed correlations between MRI parameters are driven by differences between concussed athletes with and without HOC. In future research, it will be important to examine contributions from other demographic and clinical factors, in order to more fully describe the relationships between MRI parameters.

This study has some limitations which should be considered in future research. We herein focused on relative metabolite quantification (i.e., log-ratio values), rather than absolute values. It is unclear which method provides optimal sensitivity to concussion recovery and future research should compare these approaches. In addition, the study focused on voxels localized to the primary motor cortex. Based on prior literature, different brain regions may have different neurometabolic profiles of response and microstructural change (Henry, et al., 2010; Henry, et al., 2011; Johnson, et al., 2012a), partly stemming from spatial variation in head injury biomechanics. As suggested by (Dimou and Lagopoulos, 2014), there is a need for more comprehensive assessments of SVS effects throughout the brain, to identify which regions are most sensitive to the effects of concussion. Given the apparent importance of HOC when interpreting the neurometabolic effects of concussion, more detailed long-term tracking of athletes over the course of multiple concussions is also needed to construct more detailed models of the cumulative effects of concussion on neurometabolism, and how this affects associations with other MRI parameters. Finally, there was some attrition of concussed athletes, which may lead to biased parameter estimates. Although longitudinal analyses used LMMs and cross-sectional analyses used multiple imputation techniques to mitigate these issues, more longitudinal data is needed to validate and replicate study findings.

This study is, to our knowledge, one of the most comprehensive assessments of neurometabolic recovery after sport-related concussion, evaluating changes from acute injury to one year after RTP, along with other MRI measures of brain structure and function. We identified significant, long-term neurometabolic effects of concussion which persist beyond a month post-RTP. Moreover, we showed that prior concussion history may significantly alter neurometabolic recovery after a concussion. In addition, we verified, using other advanced MRI sequences, that the identified effects at RTP are likely distinct from those at early injury and correlated with changes in white matter microstructure and brain function. These findings help to advance our understanding of concussion pathophysiology and to better characterize its relationship with clinical indices of concussion recovery.

## Funding

This work was supported by the Canadian Institutes of Health Research (CIHR) [grant numbers RN356342 – 401,065, RN294001–367,456]; the Canadian Institute for Military and Veterans Health Research (CIMVHR) [grant number W7714-145,967]; and Siemens Healthineers Canada.

## Appendix Supplementary materials

Image, application 1

Image, application 2

Image, application 3

Image, application 4
