# Supplementary material for: Neurometabolites and sport-related concussion: From acute injury to one year after medical clearance
Source: Neuroimage Clin. 2020 Apr 22;27:102258. doi: 10.1016/j.nicl.2020.102258 (PMC7215245; doi:10.1016/j.nicl.2020.102258)
Supplement: Supplementary file 1 [file mmc1.docx]

**Appendix-A: Athlete numbers by sport**

**Table A1:** athlete numbers by sport, for both male (M) and female (F) groups, for N=66 controls and N=33 concussed athletes.

| **CONTROL** | **CONCUSSION** |
| --- | --- |
| Water polo (1M)  Lacrosse (3M*)  Basketball (4F)  Rugby (8F*)  Football (3M*)  Soccer (7M / 4F)  Hockey (6M* / 10F)  Volleyball (10M / 10F) | Water polo (1M)  Lacrosse (1M* / 2F)  Basketball (1M / 2F)  Rugby (4M* / 9F*)  Football (4M*)  Hockey (4M* / 1F)  Volleyball (1M / 3F) |

* collision sports, defined as involving routine, purposeful body-to-body contact^1^

1. Meehan III, W.P., Taylor, A.M., Berkner, P., Sandstrom, N.J., Peluso, M.W., Kurtz, M.M., Pascual-Leone, A. and Mannix, R. (2016). Division III collision sports are not associated with neurobehavioral quality of life. Journal of neurotrauma 33, 254-259.
