# Supplementary material for: Neurometabolites and sport-related concussion: From acute injury to one year after medical clearance
Source: Neuroimage Clin. 2020 Apr 22;27:102258. doi: 10.1016/j.nicl.2020.102258 (PMC7215245; doi:10.1016/j.nicl.2020.102258)
Supplement: Supplementary file 2 [file mmc2.docx]

**Appendix-B: longitudinal effects of concussion using choline reference**

**Table B1**: longitudinal effects of concussion on NAA/Cho and Ins/Cho response. The table reports coefficients of fixed-effect $b$, 95% confidence intervals (95% CIs), bootstrap ratios (BSRs) and p-values. Longitudinal tests compare imaging sessions (RTP, 1MO, 1YR) to ACU along with interactions with history of concussion (HOC), days post injury (dACU) and days to RTP (dRTP).

|  | NAA/Cho | | | | Ins/Cho | | | |
| --- | --- | --- | --- | --- | --- | --- | --- | --- |
|  | *b* | 95%CI | BSR | *p* | *b* | 95%CI | BSR | *p* |
| ACU | -- | -- | -- | -- | -- | -- | -- | -- |
| RTP | 0.120 | [0.043, 0.275] | 2.75 | 0.004 | 0.244 | [0.057, 0.461] | 2.76 | 0.014 |
| 1MO | -0.075 | [-0.256, 0.166] | -0.50 | 0.574 | 0.329 | [0.148, 0.548] | 3.51 | 0.006 |
| 1YR | -0.079 | [-0.274, 0.163] | -0.56 | 0.516 | 0.090 | [-0.249, 0.548] | 0.67 | 0.474 |
| ACU:HOC | 0.020 | [-0.133, 0.164] | 0.24 | 0.810 | -0.021 | [-0.218, 0.164] | -0.25 | 0.786 |
| RTP:HOC | -0.109 | [-0.314, -0.006] | -1.94 | 0.042 | -0.317 | [-0.502, -0.163] | -3.95 | 0.000 |
| 1MO:HOC | 0.041 | [-0.208, 0.201] | 0.20 | 0.784 | -0.434 | [-0.688, -0.247] | -4.31 | 0.000 |
| 1YR:HOC | 0.157 | [-0.155, 0.399] | 1.03 | 0.282 | -0.346 | [-0.838, 0.008] | -1.78 | 0.062 |
| ACU:dACU | 0.054 | [-0.005, 0.118] | 1.58 | 0.078 | -0.034 | [-0.143, 0.041] | -1.21 | 0.226 |
| RTP:dRTP | 0.007 | [-0.075, 0.11] | 0.35 | 0.730 | -0.001 | [-0.109, 0.155] | 0.14 | 0.898 |
| 1MO:dRTP | -0.105 | [-0.187, 0] | -1.99 | 0.050 | -0.015 | [-0.137, 0.117] | -0.22 | 0.816 |
| 1YR:dRTP | -0.051 | [-0.181, 0.143] | -0.46 | 0.514 | 0.056 | [-0.253, 0.291] | 0.16 | 0.828 |

**Table B2**: cross-sectional comparison of concussed NAA/Cho and Ins/Cho response to uninjured controls. The table reports mean effects, 95% confidence intervals (95% CIs), bootstrap ratios (BSRs) and p-values. Tests compare imaging sessions (ACU, RTP, 1MO, 1YR), for athletes individuals without and with history of concussion (HOC).

|  |  | NAA/Cho | | | | Ins/Cho | | | |
| --- | --- | --- | --- | --- | --- | --- | --- | --- | --- |
|  |  | *mean* | 95%CI | BSR | *p* | *mean* | 95%CI | BSR | *p* |
| ACU | (no HOC) | 0.046 | [-0.130, 0.224] | 0.51 | 0.582 | 0.050 | [-0.166, 0.268] | 0.46 | 0.637 |
|  | (HOC) | 0.043 | [-0.101, 0.194] | 0.59 | 0.558 | 0.046 | [-0.135, 0.233] | 0.49 | 0.631 |
| RTP | (no HOC) | 0.134 | [-0.015, 0.218] | 1.74 | 0.086 | 0.295 | [0.123, 0.452] | 3.44 | **<0.001** |
|  | (HOC) | 0.050 | [-0.079, 0.181] | 0.75 | 0.440 | -0.020 | [-0.201, 0.154] | -0.22 | 0.832 |
| 1MO | (no HOC) | -0.052 | [-0.217, 0.105] | -0.65 | 0.514 | 0.371 | [0.188, 0.543] | 4.23 | **<0.001** |
|  | (HOC) | 0.004 | [-0.127, 0.132] | 0.06 | 0.970 | -0.036 | [-0.223, 0.140] | -0.38 | 0.731 |
| 1YR | (no HOC) | -0.052 | [-0.214, 0.106] | -0.63 | 0.518 | 0.141 | [-0.044, 0.325] | 1.50 | 0.126 |
|  | (HOC) | 0.106 | [-0.014, 0.229] | 1.65 | 0.100 | -0.201 | [-0.376, -0.026] | -2.26 | 0.019 |
