# Supplementary material for: Neurometabolites and sport-related concussion: From acute injury to one year after medical clearance
Source: Neuroimage Clin. 2020 Apr 22;27:102258. doi: 10.1016/j.nicl.2020.102258 (PMC7215245; doi:10.1016/j.nicl.2020.102258)
Supplement: Supplementary file 3 [file mmc3.docx]

**Appendix-C: data imputation**

Voxelwise correlational analyses were performed between neurometabolite data and other MRI data, including fractional anisotropy (FA), mean diffusivity (MD) and functional connectivity (Fconn). For each voxel analysis, we have a matrix of parameter values ($S$ subjects $\times$ $T$ time points), which are correlated against similar-sized matrix of neurometabolites values. Prior to analysis, imputation was done to replace missing values within each matrix using the SOFT-IMPUTE algorithm (Mazumder, et al., 2010). This is a matrix factorization approach to estimating missing data; these methods are appealing due to their ability to handle datasets with substantial missing data, while making minimal assumptions about the underlying data distributions (Hastie, et al., 2015; Ranjbar, et al., 2015; Zhou, et al., 2008). SOFT-IMPUTE has advantages of being computationally efficient and requiring relatively little model tuning to optimize. Imputation was performed separately for each data matrix prior to correlational analysis, to avoid bias caused by information sharing between datasets.

The SOFT-IMPUTE algorithm and implementation are summarized as follows, for a data matrix $\boldsymbol{X}$ with missing data. Given a sequence of *K* penalty values $\lambda_{1}>\ldots>\lambda_{k}>\ldots>\lambda_{K}$, we obtain an imputed solution of $\boldsymbol{X}$ for each penalty, by calculating the singular value decomposition $\boldsymbol{UD}\boldsymbol{V}^{T}=\boldsymbol{X}$ of rank *R* and replacing missing values of $\boldsymbol{X}$ with the approximating values of $\hat{\boldsymbol{X}}\boldsymbol{=U}\boldsymbol{D}^{\boldsymbol{*(k)}}\boldsymbol{V}^{T}$, where diagonal elements of $\boldsymbol{D}^{\boldsymbol{*(k)}}$ are calculated as ${\boldsymbol{D}_{ii}}^{\boldsymbol{*(k)}}\boldsymbol{=}max\left[ \left( \boldsymbol{D}_{ii}-\lambda_{k} \right), 0 \right]$; this process is repeated until estimates of $\hat{\boldsymbol{X}}$ converge. For all $k>1$, the $k^{th}$solution is also initialized using the imputed estimates from the $\left( k-1 \right)^{th}$ solution to improve model convergence. For each data matrix of interest $\boldsymbol{X}$, performance was evaluated over a penalty range of $\lambda_{1}=\boldsymbol{D}_{11}-\left( \boldsymbol{D}_{RR}\boldsymbol{/}100 \right)$ to $\lambda_{K}=\left( \boldsymbol{D}_{RR}\boldsymbol{/}100 \right)$, evaluated in 100 evenly-spaced log-scale increments, with a stopping criterion of $\left\| \hat{\boldsymbol{X}_{iter}}- \hat{\boldsymbol{X}_{iter-1}} \right\|/\left\| \hat{\boldsymbol{X}_{iter-1}} \right\|<{10}^{-6}$.

The optimal value of $\lambda_{k}$ was estimated for each data set $\boldsymbol{X}$ using a cross-validation approach: we created “simulated missing” (SM) datapoints, by removing 7 non-missing datapoints, selected at random (~10% of non-missing data), and fitting the imputation model on remaining data. We then evaluated model accuracy as a function of penalty $\lambda_{k}$ in terms of mean-squared-error (MSE) of the predicted (imputed) values given the true SM values. This was repeated for 200 iterations, and the value of $\lambda_{k}$ that minimized average MSE was held fixed for subsequent bootstrapping analyses. For computational efficiency, an optimal setting of $\lambda_{k}$ was chosen for all voxels of a given MRI parameter (FA, MD or Fconn) by performing full optimization for a randomly-selected 10% of all brain voxels and, after determining that the distribution of $\lambda_{k}$ values was unimodal by visual inspection, selecting the distribution mean.

**REFERENCES**

Hastie, T., Mazumder, R., Lee, J.D., Zadeh, R. (2015) Matrix completion and low-rank SVD via fast alternating least squares. The Journal of Machine Learning Research, 16:3367-3402.

Mazumder, R., Hastie, T., Tibshirani, R. (2010) Spectral regularization algorithms for learning large incomplete matrices. Journal of machine learning research, 11:2287-2322.

Ranjbar, M., Moradi, P., Azami, M., Jalili, M. (2015) An imputation-based matrix factorization method for improving accuracy of collaborative filtering systems. Engineering Applications of Artificial Intelligence, 46:58-66.

Zhou, Y., Wilkinson, D., Schreiber, R., Pan, R. (Large-scale parallel collaborative filtering for the netflix prize). In; 2008. Springer. p 337-348.
