# Supplementary material for: Neurometabolites and sport-related concussion: From acute injury to one year after medical clearance
Source: Neuroimage Clin. 2020 Apr 22;27:102258. doi: 10.1016/j.nicl.2020.102258 (PMC7215245; doi:10.1016/j.nicl.2020.102258)
Supplement: Supplementary file 4 [file mmc4.docx]

**Appendix-D: normal test-retest variability of neurometabolites**

We obtained estimates of intra-subject neurometabolite variability for healthy adults, as a reference for evaluating longitudinal effects among concussed athletes. Data were drawn from three multi-subject studies that that used STEAM sequences and reported intra-subject variability estimates for creatine (Cr), N-acetyl aspartate (NAA) and myo-inositol (Ins) in terms of coefficients of variation (CoVs) (Bartha, et al., 2000; Brooks, et al., 1999; Geurts, et al., 2004). For most studies, CoVs were reported for individual metabolite values; hence, we converted them into variability estimates for log-ratios log(NAA/Cr) and log(Ins/Cr). By standard propagation of error formulae, the absolute error of log-ratio measurement log(A/B), assuming a “worst-case” of independent numerator and denominator, is given by:

$$\sigma\left[ \log\left( \frac{A}{B} \right) \right]=\sigma\left[ \log\left( A \right)-\log\left( B \right) \right]=\sqrt{\left( \sigma_{A}/A \right)^{2}+\left( \sigma_{B}/B \right)^{2}}$$

Using the CoVs to approximate the error ratio terms $\sigma_{A}/A$, we obtain estimates for absolute standard deviation (SD) of the log-ratio values. These values are summarized in Table D1 below.

**Table D1**: table summarizing fractional coefficients of variance (CoVs) for individual neurometabolites based on

| **Study** | **N** | **Cr (CoV)** | **NAA (CoV)** | **Ins (CoV)** | **log(NAA/Cr) (SD)** | **log(Ins/Cr) (SD)** |
| --- | --- | --- | --- | --- | --- | --- |
| Brooks et al. (1999) | 10 | 0.043 | 0.033 | 0.081 | 0.054 | 0.092 |
| Bartha et al. (2000) | 10 | 0.057 | 0.073 | 0.124 | 0.093 | 0.137 |
| Geurts et al. (2004) | 15 | 0.055 | 0.046 | 0.045 | 0.072 | 0.071 |

Pooling variability across studies as ${SD}_{pool}=\sqrt{\sum_{i} N_{i}{{SD}_{i}}^{2}/\sum_{i} N_{i}}$, we obtain for log(NAA/Cr) an SD of 0.074 and for log(Ins/Cr) an SD of 0.100. From these estimates, we may obtain the normal 95%CIs associated with healthy brain variability, centered about zero, for log(NAA/Cr) of ± 0.145 and for log(Ins/Cr) of ± 0.196.

**REFERENCES**

Bartha, R., Drost, D., Menon, R., Williamson, P. (2000) Comparison of the quantification precision of human short echo time 1H spectroscopy at 1.5 and 4.0 Tesla. Magnetic Resonance in Medicine: An Official Journal of the International Society for Magnetic Resonance in Medicine, 44:185-192.

Brooks, W.M., Friedman, S.D., Stidley, C.A. (1999) Reproducibility of 1H‐MRS in vivo. Magnetic Resonance in Medicine: An Official Journal of the International Society for Magnetic Resonance in Medicine, 41:193-197.

Geurts, J.J., Barkhof, F., Castelijns, J.A., Uitdehaag, B.M., Polman, C.H., Pouwels, P.J. (2004) Quantitative 1H‐MRS of healthy human cortex, hippocampus, and thalamus: metabolite concentrations, quantification precision, and reproducibility. Journal of Magnetic Resonance Imaging: An Official Journal of the International Society for Magnetic Resonance in Medicine, 20:366-371.
